# Accuracy of Computed Tomography Attenuation Value of Lumbar Vertebra to Assess Bone Mineral Density

**DOI:** 10.21315/mjms2021.28.1.6

**Published:** 2021-02-24

**Authors:** Kok King Chia, Juhara Haron, Nik Fatimah Salwati Nik Malek

**Affiliations:** 1 Department of Radiology, School of Medical Sciences, Universiti Sains Malaysia, Kelantan, Malaysia; 2 Department of Radiology, Hospital Sultanah Bahiyah, Alor Setar, Kedah, Malaysia

**Keywords:** osteoporosis, post-menopausal bone loss, multidetector computed tomography, dual-energy X-ray absorptiometry, lumbar vertebrae, compression fracture, metabolic bone disease

## Abstract

**Background:**

Computed tomography (CT) attenuation (Hounsfield unit [HU]) value of lumbar vertebra may provide an alternative method in the detection of osteoporosis during CT scans.

**Methods:**

A cross-sectional study on 50 patients of age 50 and above with contrast-enhanced CT (CECT) and dual-energy X-ray absorptiometry (DXA) was conducted from November 2018 to November 2019. Single region of interest (ROI) was placed at the anterior trabecular part of L1 vertebra on CECT to obtain HU value. Correlation of CT HU value of L1 vertebra and DXA T-score, interrater reliability agreement between HU value of L1 vertebra and T-score in determining groups of with and without osteoporosis, ROC curve analysis for diagnostic accuracy and cut-off value of CT for detection of osteoporosis were identified.

**Results:**

Significant correlation between HU value of L1 vertebra and L1 T-score (*r* = 0.683)/lowest skeletal T-score (*r* = 0.703) (*P* < 0.001). Substantial agreement between HU value of L1 vertebra and DXA in determining the groups with and without osteoporosis (*k* = 0.8; *P* < 0.001). The area under the receiver operating characteristic (AUROC) curve was 0.93 (95% CI: 0.86, 1.00) using HU value (*P* < 0.001). Cut-off value for osteoporosis was 149 HU.

**Conclusion:**

HU value of lumbar vertebra is an effective alternative for the detection of osteoporosis with high diagnostic accuracy in hospitals without DXA facility.

## Introduction

Osteoporosis is a skeletal disorder where bone density and quality of bones deteriorate, eventually causing fragility fracture from minor impact and poses a significant healthcare concern in this modern society in terms of morbidity and mortality, and social burden. Osteoporosis is divided into primary and secondary osteoporosis (1). Primary osteoporosis occurs in the normal ageing process, while secondary osteoporosis is due to other etiologies. This pathological condition often affects post-menopausal women due to the decline of the hormone estrogen. Common osteoporotic fractures sites are the femoral neck, lumbar and thoracic vertebral fractures. Vertebral fractures are the commonest; however, most of the patients are asymptomatic and do not seek medical consultation (2). Unfortunately, it is often being underdiagnosed, undertreated, and neglected due to lack of awareness amongst both the public and the healthcare personnel. The International Osteoporosis Foundation (IOF) stated that 1 in 3 women and 1 in 5 men over age 50 years old may suffer from osteoporotic fractures globally. The female-to-male ratio osteoporotic fractures is 1.6 with overall 61% of fractures in women. IOF conducted a survey in 11 countries and found out that the result of underdiagnosis and undertreatment of the disease were mainly contributed by the denial of personal risks in post-menopausal women, lack of discussion on osteoporosis with doctors, and restricted access to the diagnosis and treatment of osteoporosis (3).

Dual-energy X-ray absorptiometry, also known as DXA, is the gold standard in the diagnosis of osteoporosis. DXA utilises a relatively low radiation imaging modality where two X-ray beams of different energy levels are emitted to soft tissues and bones to measure the tissue density. Scanning time is relatively short, which usually can be done within a 5 min–10 min interval. DXA scanners are available in the major cities of East and West Malaysia, with a total of 56 scanners (4). However, such service is not readily accessible to the suburban and rural areas. *The Clinical Guidance on Management of Osteoporosis 2012* published by Malaysian Osteoporosis Society stated that apart from the Klang Valley area, there is limited availability of DXA machines outside the capital towns in each state (5). Furthermore, DXA only provides 2D planar images for the quantification of the bone mineral density (BMD) and its quantification is often degraded by osteophytes, focal bone defect, sclerotic bone lesion, bone fracture, instrumentation etc.

Computed tomography (CT) is widely available in most hospitals including some minor specialist hospitals in Malaysia. Statistics in year 2018 showed a total of 245 CT scanners were available in public, private and university hospitals across the country (6). With the modern advancement of technology, currently osteoporosis can be diagnosed with quantitative computed tomography (QCT) using low dose radiation to quantify the bone density by comparing the attenuation value of the vertebral body with the density of the hydroxyapatite on the phantom. Unfortunately, most of the CT scanners in Malaysia are not equipped with such modern imaging methods due to its higher cost.

Contrast-enhanced CT (CECT) scans are frequently requested in the radiology department for diagnosis and response monitoring for various disorders, ranging from simple appendicitis to complex cancers. In CECT, intravenous (IV) contrast media are often given to enhance the contrast between tissues. In pathological condition, tissues with increased vascularity typically display vivid enhancement following IV contrast media administration. CECT scans are sometimes complemented by the non-contrasted scan and delayed scan to assess the pattern of enhancement, usually in the imaging of cancers.

CT images are reconstructed based on the attenuation of X-ray and the complex mathematical calculation of the attenuation coefficient. With the attenuation coefficient calculated, images are formed based on the voxels. Hence, volumetric images can be generated. CT attenuation value, also known as Hounsfield unit (HU) value, is used to determine the density of the irradiated tissues. HU is the linear transformation of the linear attenuation coefficient in determining the density of tissues where the HU of water at standard room and temperature (STP) is 0 while air is −1000.

The trabecular bone loss in osteoporotic bones causes a reduction in bone mineral density. This leads to a lucent and hypoattenuating appearance on X-ray imaging. However, without the equipment of sophisticated imaging methods such as DXA or QCT, proper quantification of bone loss cannot be accurately determined. CT HU value may help to determine the bone mineral density (BMD) loss by placing the region of interest (ROI) at the bone trabeculae, where dense bones usually measure from +700 HU to +3000 HU.

A concurrent screening for patients with CECT scan may offer a crucial role in the detection of osteoporosis. A more straightforward method of BMD assessment on CT scans can be made by measuring the CT HU value of the trabeculae of the vertebral body. According to studies conducted by Pickhardt et al. (7, 8), CT was used to compare the ability to assess BMD with DXA. The studies were conducted in non-contrast CT scans and CECT scans. There was a promising result in the correlation between the CT and DXA to detect osteoporosis.

Hence, the rationale of this study is to correlate the HU value of the trabecula of the lumbar vertebrae with the bone mineral density (T-score) on DXA. This can be easily achieved by placing the ROI at the trabecular part of the vertebral body in a CECT scan. This method will significantly help radiologists in making the diagnosis of osteoporosis without subjecting patients to DXA scan. This method can primarily assist in decision making of initiation of calcium or vitamin D supplements to prevent the occurrence of osteoporotic fractures.

The objective of this study is to determine the accuracy of HU value for BMD assessment. The correlation between CT and DXA and the diagnostic accuracy of CT HU value for the assessment of BMD are studied. We hypothesise that CT could accurately assess BMD at the L1 vertebra as compared to DXA.

## Methods

### Patient Population and Selection

A cross-sectional study that was approved by the Human Research Ethics Committee of Universiti Sains Malaysia (USM/JEPeM/18050229) and National Medical Research Register (NMRR, research ID 39076) was conducted at Hospital Sultanah Bahiyah (HSB), Alor Setar, Malaysia for a period of 12 months from November 2018 to November 2019. All patients aged 50 years old and above who underwent CECT scans for any medical conditions including lumbar vertebrae were eligible. Patients with pre-existing L1 vertebral fracture, lumbar deformity, severe degenerative disease, spinal instrumentation, vertebroplasty and immobility were excluded from the study. CECT scans were chosen because non-contrast CT scans are not routinely performed in our centre for detection of neoplastic lesions. Those who consented for the study were subjected to DXA examination within 3 weeks to 6 months after the CT. Sample size estimation to correlate between mean HU value of L1 vertebra on CECT and BMD of lumbar vertebrae on DXA was calculated using Sample Size Calculator for Correlation Analysis version 3.0. Prior study indicated that the correlation coefficient between mean HU value and BMD was 0.48 (9). Hence, a minimum sample size of 32 samples were needed to be able to reject the null hypothesis with probability (power) 0.8. The Type I error probability associated with this test of this null hypothesis was 0.05. With an additional of 10% dropout rate, the corrected sample size was 36 samples. While the sample size estimation for diagnostic accuracy was calculated based on the prior study (7). Previous data indicated that an area under the receiver operating characteristic (ROC) curve of 0.830 for CT performance in predicting osteoporosis was significant from the null hypothesis value 0.6. Hence, a minimum sample size of 40 samples were needed to be able to reject the null hypothesis with probability (power) 0.8. The Type I error probability associated with this test of this null hypothesis was 0.05. With an additional 20% dropout rate, the sample size was 48 samples. No sampling method was applied because all eligible patients that fulfilled the inclusion criteria were enrolled in the study.

### Computed Tomography

#### CT Acquisition Technique

All CECT scans were performed on Siemens multidetector CT scanner (Somatom Emotion, Siemens, six slices), utilising a standard 120 kVp setting. Variable milliampere (mA) was utilised according to scan protocol because it did not affect the HU value. Each CECT scan protocol was different according to the indications, but vast majority of post-contrast series were obtained during the portal venous phase (approximately 60 sec after contrast injection). Axial images were acquired with thin collimation reconstructed with 5.0 mm thickness at 3.0 mm intervals using a standard soft tissue algorithm. A routine coronal and sagittal reconstructions were obtained with 5.0 mm thickness at 2.5 mm intervals. The axial CT series were assessed on a standard radiology picture archiving and communication system (PACS) workstation. In this study, CECT scans were chosen because, in many diagnostic centers, non-contrast CT scans are not routinely performed for detection of neoplastic lesions. Contrast media was given to characterise any lesions for diagnostic purposes.

#### Vertebral Osseous Assessment

Vertebral assessment was performed using Centricity PACS RA1000, GE Healthcare workstation with images viewed in bone window setting as follow:

- window width (W) = 3077
- window level (L) = 570

The L1 vertebra, the fifth vertebra counted cranially from the last unfused vertebra, which was taken as L5 vertebra, or the first vertebra counted caudally from the last vertebra with rib which was taken as T12 vertebra, was chosen due to easy identification as the first non-rib-bearing vertebra and included on all thoracic, abdominal and pelvic CT scans in clinical practice since it provides the best overall results in terms of DXA correlation (7).

**Figure 1:**
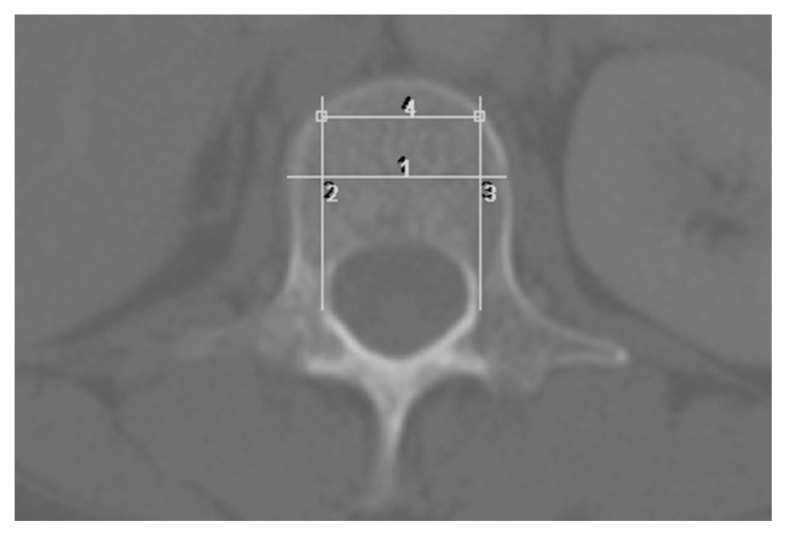
Method of drawing ROI. A rectangular box in the trabecular part of the bone. [1] Line dividing the vertebral body into anterior and posterior halves; [2] & [3] Parallel lines from both pedicles intersecting with the first line; [4] Line connecting the two parallel lines at the level of the inner table of the vertebral body

**Figure 2:**
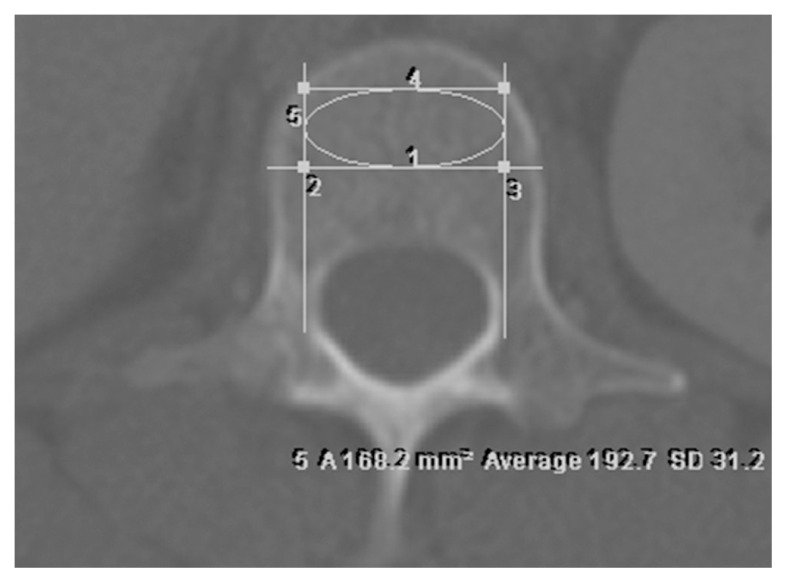
Ovoid ROI in the rectangular focus. An ovoid ROI is drawn within the rectangular focus with area of ROI, HU value and standard deviation (SD) displayed. [1] Line dividing the vertebral body into anterior and posterior halves; [2] & [3] Parallel lines from both pedicles intersecting with the first line; [4] Line connecting the two parallel lines at the level of the inner table of the vertebral body; [5] Ovoid ROI

The area of measurement should be centred at the anterior region of the vertebral body, excluding the cortical margins. Care should be taken to avoid the posterior venous plexus, focal heterogeneity or any imaging-related artefacts which may produce overestimation or underestimation of the HU value. To avoid erroneous results, a rectangular area was created by drawing a line dividing the vertebral body into anterior and posterior halves. Next, two parallel lines both pedicles intersecting perpendicularly with the first line. Another line was drawn connecting the two parallel lines at the level of the inner table of the compact bone of the vertebral body. These lines formed a rectangular ROI in the trabecular part of the vertebra (Figure 1). A single ovoid ROI was placed within the drawn area for measurement of mean HU value. The area of ROI was limited to an area between 150 mm^2^–200 mm^2^ for standardisation (Figure 2).

Total mean HU value of three parts of the L1 vertebra were measured as follow and taken for analysis:

- immediately inferior to the superior endplate
- mid vertebral body
- immediately superior to the inferior endplate

This simple ROI attenuation method did not require a phantom, oblique angulation along the disc plane, multi-level assessment, or ROI placement in muscle and fat as may be performed in QCT. It was also relatively simple and required minimal time and effort for the radiologist to interpret the results.

#### Dual-Energy X-ray Absorptiometry and BMD Assessment

DXA scans were performed on the lumbar spine from the first to the fourth lumbar vertebrae (L1–L4) and proximal femur using standard techniques (Horizon DXA System, HOLOGIC). The skeletal sites that were assessed include L1–L4 vertebrae, total proximal femur and femoral neck. T-score of each skeletal site was generated. For this study, the L1 T-score and the lowest skeletal T-score were taken for correlation with CT HU value for BMD assessment.

The WHO’s classification of BMD was used to categorise patients into normal, osteopenia and osteoporosis using T-score (10). The lowest skeletal T-score of the lumbar vertebrae or proximal femur was used to determine diagnosis (11):

- normal: T-score ≥ −1.0
- low bone mass (osteopenia): −2.5 < T-score < −1.0
- osteoporosis: T-score ≤ −2.5
- severe (established) osteoporosis: T-score ≤ −2.5 with one or more fragility fractures

#### Statistical Analysis

All data were analysed using Statistical Product and Service Solutions (SPSS) for Windows, SPSS Inc.© (version 24, SPSS Inc., Chicago, IL, USA). The descriptive statistics for discrete variables (gender) were presented as *n* = frequency (%) and the continuous variables (HU value of osteoporosis, low bone mass and normal BMD) were presented as mean, standard deviation and 95% CI. Comparison between DXA T-score and CT HU value in BMD assessment was done using one-way ANOVA.

Pearson’s correlation was used to correlate CT HU value of L1 vertebra with the DXA T-score of L1 vertebra and the lowest skeletal site. Interrater reliability for the agreement between the HU value of L1 vertebra and DXA in determining the group of no osteoporosis and osteoporosis was evaluated using Cohen’s kappa analysis. Diagnostic accuracy of CT in detection of osteoporosis and its cut-off HU value were determined using ROC curve analysis. The cut-off value was selected from the coordinates of the curve that yielded the highest true positive rate (TPR) and lowest false positive rate (FPR), which was translated into highest sensitivity and specificity.

## Results

A total of 50 subjects with CECT scans which included the lumbar vertebrae consented for the participation of this study. There were 94% (*n* = 47) of females and 6% (*n* = 3) of males. Among the 50 subjects, 26% (*n* = 13) had low bone mass and 50% (*n* = 25) had osteoporosis. The rest of the subjects had normal BMD. The HU value at L1 vertebra was measured in the ROI and yielded a mean (SD) of 108.29 HU (25.60) [95% CI: 98.36–118.21] for osteoporosis, 164.27 HU (13.42) [95% CI: 157.37–171.18] for low bone mass, and 212.07 HU (12.94) [95% CI: 196.00–228.13] for those with normal BMD on one-way ANOVA (Table 1).

The correlation of HU value with the DXA T-score of L1 vertebra and the lowest skeletal site was significant in determining osteoporosis. The analysis showed Pearson’s correlation coefficient (*r*) of 0.683 for L1 T-score and 0.703 for lowest T-score (*P* < 0.001) (Table 2).

Interrater reliability using Cohen’s kappa analysis showed a substantial agreement between HU value of L1 vertebra and T-score in determining the groups with osteoporosis and no osteoporosis, with a Cohen’s kappa coefficient of 0.8 (*P* < 0.001) (Table 3).

**Figure 3:**
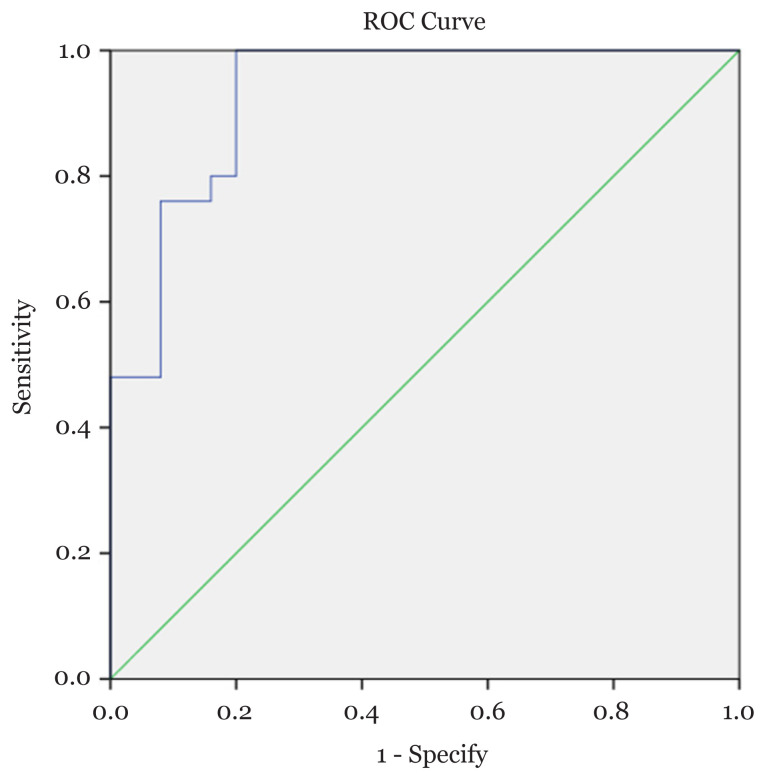
ROC curve for diagnostic accuracy of CT in the detection of osteoporosis. AUC 0.93 (95% CI: 0.86, 1.00) (*P* < 0.001)

To discriminate between the groups of osteoporosis and no osteoporosis using CT HU value, the area under the curve (AUC) in ROC curve analysis yielded an excellent result of 0.93 (95% CI: 0.86, 1.00) (Figure 3), which was statistically significant (*P* < 0.001). In other words, the CT HU value can accurately distinguish 93.4% of cases between the group of osteoporosis and no osteoporosis with a sensitivity and specificity of 80% and 100%, respectively. The data were shown in Table 4. The best cut-off HU value of L1 vertebra for maintaining the highest sensitivity and specificity was determined at 149 HU, as shown in Table 5.

## Discussion

Our study showed a significant correlation between the mean HU value of L1 vertebra and T-score of L1 vertebra, as well as the lowest skeletal T-score with correlation coefficient of 0.683 and 0.703, respectively, in determining osteoporosis. Significant correlations between the mean HU and T-score of L1 vertebra were found in previous studies with correlation coefficients of 0.573 and 0.708 (11, 12). We utilised the lowest skeletal T-score in our study due to highest correlation with DXA BMD for the diagnosis of osteoporosis. There was a substantial agreement on Cohen’s kappa analysis in using the mean CT HU and DXA BMD to detect patients with and without osteoporosis (κ = 0.8).

We included 25 patients with osteoporosis with mean (SD) HU of L1 vertebra of 108.29 HU (± 25.60) [95% CI: 98.36–118.21]. We found that the mean cut-off HU value at L1 vertebra of 149 HU (sensitivity 80%, specificity 100%) able to yield a high diagnostic accuracy to distinguish between osteoporotic and non-osteoporotic groups. The AUC value on the ROC curve in our study was 0.93. Several other studies have reported significant diagnostic accuracy of HU value on lumbar vertebra. Pickhardt et al. (7) reported a high diagnostic accuracy with an AUC value of 0.83 in a cohort of 157 adults. Alacreu et al. on 111 osteoporotic Southern European patients reported an AUC value of 0.664 (13). The cut-off values for the diagnosis of osteoporosis for the two studies were 144 HU (sensitivity 90%, specificity 64%) and 160 HU (sensitivity 90%), respectively.

Statistics showed higher prevalence of osteoporosis in women aged 50 years old and above, worldwide, which accounts for 40% in Germany, 11.5% in Romania, 25% in Argentina, 50.1% in China, 29% in India and so on (3). In Malaysia, the population aged over 50 years old is expected to increase from 5.3 million in 2013 to a staggering 13.9 million in 2050 (3). While the incidence of hip fracture is expected to increase 3.55-fold by the year 2050 (14). Amongst the races, Chinese is the most vulnerable group recording the highest incidence of hip fracture (15). The overall prevalence of osteoporosis among the post-menopausal women in central Malaysia is 24.1% (16). Our study population reflected a higher percentage of osteoporosis than reported by Subramaniam S et al. where 50.0% of patients were osteoporosis.

We acknowledge several limitations of our study. This was a single-centre retrospective study. The sample size was small, partly due to the lack of awareness for the existence of osteoporosis. Our data were taken from Malaysian population. The modifiable and non-modifiable risk factors, such as lifestyle and underlying illnesses, were not assessed in this study. Our study is not aimed to replace DXA as the gold standard for the diagnosis of osteoporosis. However, this could be an alternative methodology to detect osteoporosis in the absence of DXA facility. Fatal complications can be prevented by initiating the treatment in early stage.

## Conclusion

There was a significant correlation between HU value of L1 vertebra and DXA BMD value with high accuracy in the detection of osteoporosis. The cut-off value of 149 HU at L1 vertebra able to detect osteoporosis in adults aged 50 and above. The mean HU value of L1 vertebra may provide an alternative in detection of osteoporosis in hospitals without the facility of DXA. This method of drawing a single ROI at the anterior trabecular part of L1 vertebra was easy and required little effort from the radiologists without any extra radiation. By employing this technique, the rate of detection of osteoporosis could be improved.

## Figures and Tables

**Table 1:** Comparison between DXA T-score and CT HU value in BMD assessment

		Mean (SD)		

		Osteoporosis (*n* = 25)	Low bone mass (*n* = 13)	Normal (*n* = 12)
DXA	L1 T-score	−2.60 (0.63)	−1.53 (0.51)	0.03 (0.97)
	Lowest skeletal T-score	−3.16 (0.43)	−1.87 (0.41)	−0.4 (0.64)
CT	HU value	108.29 (25.60)	164.27 (13.42)	212.07 (12.94)

**Table 2:** Correlation between mean CT HU value and DXA T-score for osteoporosis (*n* = 25)

DXA site	*r*	*P*-value
L1 T-score	0.683	< 0.001
Lowest skeletal T-score	0.703	< 0.001

Note: *r* = Pearson’s correlation coefficient

**Table 3:** The agreement between CT HU value and DXA T-score using Cohen’s kappa (κ)

	Test	T-score				
	
		No osteoporosis	Osteoporosis	n	κ	P-value
HU value	No osteoporosis	20	0	20	0.8	< 0.001
	Osteoporosis	5	25	30		
	*n*	25	25	50		

**Table 4:** Cut-off value, sensitivity and specificity for osteoporosis based on ROC curve analysis

Cut-off value for osteoporosis	149 HU
Sensitivity for osteoporosis	80% (20/25)
Specificity for osteoporosis	100% (25/25)

**Table 5:** Cut-off HU value determination based on coordinates of ROC curve analysis

Mean HU	Sensitivity (TPR)	1 – Specificity (FPR)
43.666	.000	.000
49.666	.040	.000
62.833	.080	.000
76.000	.120	.000
82.500	.160	.000
86.333	.200	.000
89.166	.240	.000
92.000	.280	.000
94.833	.320	.000
97.333	.360	.000
100.333	.400	.000
102.500	.440	.000
106.500	.480	.000
112.666	.480	.040
116.333	.480	.080
117.666	.560	.080
119.833	.600	.080
122.000	.640	.080
123.666	.680	.080
126.666	.720	.080
129.166	.760	.080
129.666	.760	.120
131.666	.760	.160
133.833	.800	.160
135.833	.800	.200
139.000	.840	.200
142.666	.880	.200
145.333	.920	.200
147.000	.960	.200
149.000	1.000	.200
150.833	1.000	.240
153.166	1.000	.280
156.333	1.000	.360
158.166	1.000	.400
162.333	1.000	.440
166.666	1.000	.560
167.833	1.000	.600
175.000	1.000	.640
183.166	1.000	.720
187.000	1.000	.760
189.833	1.000	.800
200.666	1.000	.840
212.833	1.000	.880
217.833	1.000	.920
222.000	1.000	.960
224.333	1.000	1.000

Notes: TPR = true positive rate; FPR = false positive rate

The smallest cut-off value is the minimum observed test value minus 1, and the largest cut-off value is the maximum observed test value plus 1. All the other cut-off values are the averages of two consecutive ordered observed test values.
